# Molecular and Serological Prevalence of *Leptospira* spp. in Feral Pigs (*Sus scrofa*) and their Habitats in Alabama, USA

**DOI:** 10.3390/pathogens9100857

**Published:** 2020-10-20

**Authors:** Anil Poudel, Md Monirul Hoque, Steven Madere, Sara Bolds, Stuart Price, Subarna Barua, Folasade Adekanmbi, Anwar Kalalah, Steven Kitchens, Vienna Brown, Chengming Wang, B. Graeme Lockaby

**Affiliations:** 1 College of Veterinary Medicine, Auburn University, Auburn, AL 36849, USA; azp0012@auburn.edu (A.P.); mzh0130@auburn.edu (M.M.H.); pricesb@auburn.edu (S.P.); szb0116@auburn.edu (S.B.); fsa0004@auburn.edu (F.A.); aak0016@tigermail.auburn.edu (A.K.); srk0002@auburn.edu (S.K.); 2 School of Forestry and Wildlife Sciences, Auburn University, Auburn, AL 36830, USA; ssm0042@auburn.edu (S.M.); szb0132@auburn.edu (S.B.); 3 USDA/APHIS/Wildlife Services, National Feral Swine Damage Management Program, Fort Collins, CO 80521, USA; vienna.r.brown@usda.gov

**Keywords:** pathogenic *Leptospira*, feral pigs, PCR, ELISA, USA

## Abstract

Leptospirosis is a widespread zoonosis and has been recognized as a re-emerging infectious disease in humans and a variety of wild and domestic animal species. In order to understand the prevalence and diversity of *Leptospira* spp. in feral pig populations of Alabama, we trapped 315 feral pigs in Bullock County east-central Alabama, and collected 97 environmental samples from riparian areas in Bullock County and Macon County east-central Alabama. Two previously published PCRs followed by DNA sequencing and BLASTn were performed to identify pathogenic *Leptospira* species in the kidney of feral pigs (3.2%, 10/315) as well as environmental samples collected from the habitats of feral pigs (2.1%, 2/97), but not in the whole blood samples (n = 276) or spleen (n = 51). An ELISA determined that 44.2% of serum samples (122/276) were antibody-positive for *Leptospira*. The identification of two pathogenic *Leptospira* species from environmental samples and the high sero-positivity in feral pigs suggests potential pathogen shedding from feral pigs to environments, and to humans and domestic animals. In order to better understand the risk to human health associated with feral swine presence, further studies are warranted to explore the interrelationship between *Leptospira* spp. shedding in the urine of feral pigs and bacterial culture to explore pathogenicity. Multi-locus sequencing typing (MLST) and microscopic agglutination tests (MAT) should be performed in future studies to make a definite determination of pathogenic *Leptospira* in feral pigs in Alabama.

## 1. Introduction

Leptospirosis is a bacterial disease of global importance that affects humans and a variety of wild and domestic animal species. It causes major economic losses in farm animals and causes significant illness and mortality in humans as well. Worldwide, approximately 500,000 human cases are reported each year with a mortality rate between 5–20% [1]. This disease is zoonotic and may be transmitted to humans along several pathways, including direct contact with water contaminated by urine from infected animals. In addition, water recreationists and campers can become infected as a result of contact with contaminated streams, rivers, and lakes [2,3,4,5].

However, there have been few rigorous studies that examined the environmental phase of *Leptospira* [2]. Leptospirosis is primarily associated with rural areas although human outbreaks have occurred in urban environments as well. In rural environments, livestock and various wildlife species (raccoons, small mammals, and bats) may serve as a reservoir while rats and dogs are the most common hosts in urban environments [4]. *Leptospira* have been classified genetically into eight pathogenic genomospecies and serologically into more than 250 serovars. Three pathogenic species known to infect swine are *Leptospira interrogans* (serovars *pomona, icterohaemorrhagiae, canicola*, and *bratislava*), *L. borgpetersenii* (serovars *sejroe* and *tarassovi*) and *L. kirschneri* (serovar *grippotyphosa*). Serovars *Pomona* and *Bratislava* are uniquely adapted to swine while others are maintained in other species but sometimes infect swine [5]. *L. interrogans* serovar *hardjo* is the most important serovar of bovines and but also infects pig in close contact with cattle. *L. interrogans* serovar *Bratislava* is the most common strain in swine, though role of this serotype as a cause of disease is debated [6]. Pathogenic *Leptospira* produce biofilm, which helps their survival in the environment, such as in soil and water distribution systems. Bacterial aggregates morphologically similar to biofilms have been identified within the lumen of proximal renal tubules of reservoir hosts, potentially making them persistent and resistance to antibiotics [7,8].

Feral pigs are considered to be the single most invasive animal species in the United States and have expanded from 17 to 38 states in the last 30 years [9]. The home range of feral swine has also spread to all 67 counties in Alabama [10]. Wild pigs have a very high rate of fecundity which poses great challenges with regard to efforts of preventing disease transmission through vaccination and the elimination of hosts as well as interventions related to the environmental conditions [3].

Although warnings of the leptospirosis risk associated with wild pigs have been issued for some parts of the United States, actual data regarding the magnitude of risk are scarce and primarily emanate from laboratory studies. Chatfeld et al. (2013) [11] found that 33% of the 324 wild pigs sampled in Florida carried antibody to one or more serovars of *Leptospira*. Similarly, Buchholz et al. (2016) [12] estimated that 33.8% of 804 feral pigs sampled in Hawaii were antibody-positive to *Leptospira* serovars. This work suggests that such a high prevalence warranted a further investigation to better describe the role of wild pigs in the etiology of leptospirosis.

On a larger scale, Pedersen et al. (2015) [13] tested whole blood from 2055 wild pigs collected across the United States for antibody presence to six strains of *Leptospira* known to infect humans and domestic animals. They found that 13% of the samples tested positive for at least one strain, and concluded that *Leptospira* infection in wild pigs is common. However, Pedersen et al. (2017) [14] observed serovar antibodies in 53% of the 677 wild pigs sampled but noted that *Leptospira* DNA was found in only 3.4% of the associated kidneys. While this situation may imply a reduced risk, the authors emphasized the need for additional research in order to fully clarify the risk to human health posed by wild pigs.

While *Leptospira* serovars have been documented to be present in significant proportions of wild pig populations in the previously mentioned studies, there remains a critical lack of understanding of the nature of transmission risk to human health and the domestic swine population posed by *Leptospira*-infected feral swine. While Kaller et al. (2015) [15] recorded the presence of leptospires in surface waters within the Kistachie National Forest in western Louisiana, their data did not reveal spatial linkages between *Leptospira* and the presence of feral pigs.

Human risk of this disease is strongly linked to environmental conditions and is higher in the tropics due to elevated humidity and temperature. Risk and presence of *Leptospira* may also be associated with neutral to alkaline soils and water, temperatures from 4 °C to 40 °C, and soil moisture content above 20% [2]. It is currently assumed that pathogenic *Leptospira* can survive in the environment but do not multiply [16]. The bacterium can survive for about a week in moist soil and mud when temperature and other conditions are favorable [3], although Thibeaux et al. (2017) [16] found that pathogenic leptospires remained viable in soils for several weeks under laboratory conditions. Emerging evidence also supports the hypothesis that rainfalls re-suspend *Leptospira* with soil particles, which are carried to surface water, where animals and humans get exposed [17].

The main objectives of this study were to examine the prevalence of leptospirosis in feral swine populations in Alabama, and examine the presence of pathogenic *Leptospira* in stream water and stream bed sediments as the habitats of feral pigs, including a privately owned tract of land and a national forest.

## 2. Results

### 2.1. Sample Compositions

We collected whole blood and tissue specimens from a total of 315 feral swine at the privately owned land (POL), with paired whole blood and kidney specimens from 276 feral pigs. A total of 77 water samples were collected from the POL, and 20 were collected from Tuskegee National Forest (TNF) (Table 1).

### 2.2. Molecular Detection of Leptospira Species

PCR and DNA sequencing identified *Leptospira* DNA in 3.2% of the kidney tissues (10/315), 2.1% of environmental samples (2/97), but none of the 276 whole blood samples or 51 spleen samples. DNA sequencing of the qPCR products identified *L. interrogans* and *L. noguchii* in pigs at POL. *L. interrogans* was identified in one soil sediment sample from the TNF (1/20), and *L. noguchii* was found in one stream water sample from the POL (1/77).

The nucleotide sequences of *L. interrogans* identified in this study (GenBank Accession #: MT447735) are identical to each other and show a full match to *L. interrogans* serovar Hardjo strain RTCC 2810 (KC800991) and serovar Gripotyphosa strain Moskva (EU871723). The nucleotide sequences of *L. noguchii* identified in this study (MT447736) are identical to each other and show a full match to that of *L. noguchii* strain LSU2580 (AY461919) and of the strain 1011 (AY461918).

### 2.3. Detection of Anti-Leptospira IgG Antibody

The uniformity of the optical density (OD) values was indicated by the control OD values that were within the acceptable range provided by the kit manufacturer and narrow 95% confident interval for substrate blank control (0.091 ± 0.001), negative control (0.097 ± 0.004), cut-off control (1.042 ± 0.023) and positive control (2.713 ± 0.075).

The porcine anti-*Leptospira* IgG antibody ELISA determined that 44.2% of serum samples (122/276) were positive. The OD value detected in samples ranged from 1.1–6.8 fold higher than the cut-off OD with an average of 2.1-fold. Forty seven out of 276 (17.0%) serum samples had an OD value at least twofold higher than the cut-off value, including 4.3% with an OD twofold higher. The highest percentage seropositive serum samples were from the month of July, followed by August and September. The monthly normalized OD ranged from a low of 1.4-fold in January 2020 to a high of 2.5-fold in September 2019.

Anti-*Leptospira* IgG antibodies were detected positive for the ten pigs that were also positive for *Leptospira* DNA by PCR (Table 2). Interestingly, the positivity of *Leptospira* IgG in feral pigs trapped in March 2020 was found to be significantly lower than those in July to September of 2019 (Table 2). Seasonal differences in the prevalence of *Leptospira* antibodies might be due to the change in the availability of plant foods and anthropogenic pressure in streams and lakes [18].

## 3. Discussions

In this study, we identified DNA of *L. interrogans* and *L. noguchii*, the two pathogenic species known to cause leptospirosis in human and animals, in kidneys from feral swine. We also identified both species from environmental samples suggesting the role of the environment in the spread of this pathogen. Strains of *L. noguchii* have been described infecting cattle [19,20,21,22] rats [23,24] and bats [25]. Apart from animal infections, the zoonotic aspect of this species has been suggested. A study in Nicaragua correlated *L. noguchii* infection in domestic animals with cumulative incidence of human cases [26], and severe clinical human cases were reported in Brazil [27]. Molecular epidemiology related to *L. noguchii* by Loureiro et al. [28] revealed important insights into the One Health context of this pathogen.

In contrast to a low number of detections of *Leptospira* DNA in feral pigs (blood: 0/276; spleen: 0/51; kidney: 10/345, 3.2%), we observed a 44.2% sero-positivity in these same animals. The discrepancy in PCR positivity and sero-positivity may be due to differences in the infectious state of the *Leptospira* spp. *Leptospira* infection typically either resolves or colonizes several target organs in the host, and disappears rapidly from circulation [29]. Chronic and asymptomatic *Leptospira* infections have been reported in several hosts [19,20,21,22,23,24,25]. The dissemination in blood usually precedes urinary shedding and colonization of kidney and spleen. Additionally, while PCR used in this study targets mainly the pathogenic *Leptospira* spp., the anti-*Leptospira* IgG ELISA detects antibodies against the infections of pathogenic and saprophytic *Leptospira* spp. This may partly explain the strong differences in the *Leptospira* positivity by PCR and ELISA.

Furthermore, the highly similar temporal distribution of normalized OD values in ELISA indicate that these pigs are constantly exposed to *Leptospira* species in the environment, and there is a real risk for cross-species transmission to wildlife species, livestock, and humans. Cross-species disease transmission between wildlife, domestic animals, and humans is an increasing threat to public and veterinary health [30,31]. Human leptospirosis occurs from indirect environment-mediated exposure to pathogenic leptospires through contaminated watered environments, possibly from the urine of infection animals, including feral pigs. The two pathogenic species of *Leptospira* detected in this study have been identified in several animal hosts in various parts of the world. Both of these *Leptospira* species were also detected in environmental samples (soil sediments and stream water), further corroborating the survival of leptospires in soil and sediments, and their role in transmission through surface water. Pathogenic *Leptospira* are known to produce biofilm that enables them to adapt to various environmental challenges [7,10]. The ability of pathogenic leptospires to survive in the aqueous environment is a key factor in transmission to new hosts [7]. *L. noguchii* was identified from sediment collected from the sites where pigs were trapped while *L. interrogans* was detected in stream water from our control site at TNF.

While leptospirosis is presumed to be the most widespread zoonoses worldwide, this disease at its onset is often misdiagnosed as a fever of unknown origin, aseptic meningitis, or influenza infection [32]. The microscopic agglutination test (MAT), showing a high sensitivity and allowing for the detection of serogroup specific antibodies [33], is the gold standard of the sero-diagnosis for leptospirosis. The IgG ELISA used in this study cannot differentiate the antibodies from infections between pathogenic and saprophytic *Leptospira*, or between different pathogenic *Leptospira* serovars. MAT should be used in the future studies to provide more definite sero-diagnosis of *Leptospira* in feral pigs in Alabama.

It is well-recognized that SYBR Greene PCR often confers false-positive results [34]. In this study, the highly specific (targeting pathogenic-*Leptospira lig* gene) and sensitive (a single copy of the target) FRET-PCR was performed to identify pathogenic *Leptospira* spp. [35]. Then, SYBR Greene PCR with a long amplicon [36] was run on those pathogenic *Leptospira*-positive samples, followed by DNA sequencing and BLASTn to identify the *Leptospira* species. The choice of using two PCRs was essential to identify pathogenic *Leptospira* spp., and minimize the workload in running PCR and performing DNA sequencing.

A Multilocus Sequence Typing (MLST) Scheme was successfully established to discriminate seven pathogenic *Leptospira* species [37]. Cilia et al. performed MLST to confirm the circulation of Tarassovi and Bratislava serogroups within wild boar in Tuscany [5]. The limitation of this study is that the determination of *Leptospira* species by PCR and DNA sequencing were not fully definite, and the MLST should be performed to make the definite determination of pathogenic serovars in the future study.

In conclusion, our PCR and serological data indicate that feral pigs present a risk of cross-species transmission of leptospirosis and may serve as a reservoir for this bacterium. The 3.2% positivity based on PCR and 44.2% sero-positivity is very similar to a study conducted by Pedersen et al. in 129 counties of 29 states, including Alabama [13,14]. This and other studies underscored the difficulty in undertaking the true epidemiological investigation of *Leptospira* infection. Studies including the culture of the organism, MLST and diagnostic assays such as MAT would be required to fully understand the risk of cross-species transmission, especially to humans and domestic animals. A longitudinal study covering a wider area would be necessary to fully understand the influence of pig density in *Leptospira* infection cycle and the associated public health risk.

## 4. Materials and Methods

### 4.1. Study Areas

The experimental procedures in this study were approved by the Auburn University Institutional Animal Care and Use Committee (Approval number: 2017-3143).

**Figure 1:**
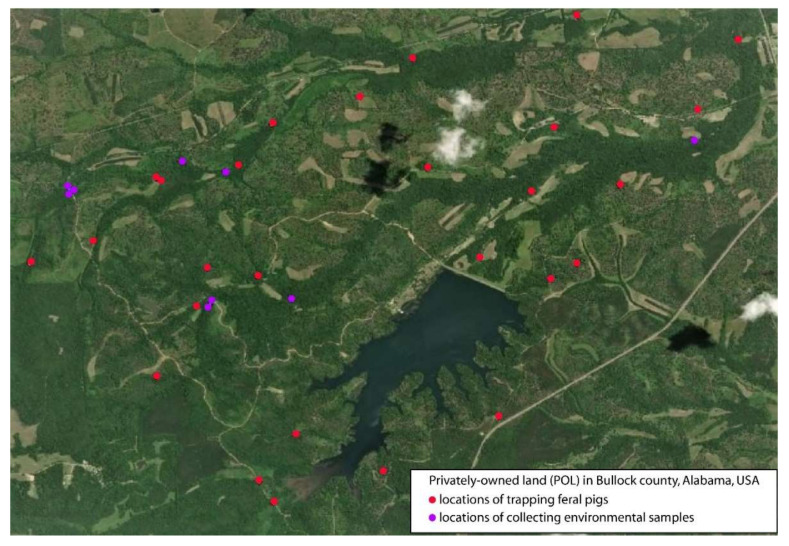
**Locations at privately owned tract of land (POL) to trap feral pigs in this study**. Feral pigs were trapped at a privately owned tract of land (POL) in Bullock County, 4515 hectares in east-central Alabama (latitude: 32.16857–32.70600 and longitude; 85.50155–85.57337). Feral pigs were trapped in 27 locations (red dots) while environmental samples were collected in 9 locations (purple dots). This figure was generated by the use of the software ArcMap 10.7.1(Redlands, CA, USA)

This study was conducted at a privately owned tract of land in Bullock County (4515 hectares) [Figure 1] and the Tuskegee National Forest located in Macon County (4451 hectares) in east-central Alabama (latitude: 32.16857–32.70600 and longitude; 85.50155–85.57337). Both properties lie within the Upper Coastal Plain physiographic region and the Mantachie–Iuka–Bibb soil association.

The POL was managed for white-tailed deer (*Odocoileus virginianus*) and eastern wild turkey (*Meleagris gallopavo silvestris*) populations, and was dominated by mixed pine (*Pinus* spp.) hardwood forest and riparian hardwoods. An earlier study at the private property estimated the wild pig density to be 15.5 pigs/km^2^, which is greater than the average density of 6–8 pigs/km^2^ in the region [38].

The study area at TNF was located approximately 25 km from the private property and was similar in terms of hydrology, watershed size, habitat type, and species composition. Wild pigs were present in the Tuskegee National Forest but were not yet established in the area selected for the study, which was confirmed with camera surveys.

Watersheds at both properties were surveyed to see if they met certain criteria: low gradient, occupied by deciduous wetland forests, and streams third order or lower streams that did not drain a standing body of water (e.g., a pond or lake). Eleven watersheds at the POL and three watersheds at the TNF were identified to be included in this study as they met the selection criteria. Floodplains at all watersheds were relatively flat with little to no riparian vegetarian buffer and soils were mainly composed of sandy bed loads. Stream flow was intermittent with peak flow occurring from November to April and greatly reduced flow during the summer months. Wild pig damage (rooting, digging, and wallowing) was observed at all selected watersheds on the POL, and occurred on the floodplains and within the stream channels throughout the year.

Water and stream sediment samples were collected at the 14 identified watersheds, while pigs were trapped at locations on the property that had frequent pig activity, which was determined by the amount of damage and number of pig sightings.

### 4.2. Trapping of Feral Pigs and Collection of Tissues

Between July 2019 and March 2020, 315 feral pigs were trapped in this study as part of a feral swine removal program funded by the USDA-APHIS. The pigs were captured using Jager Pro Hog Control Systems corral trap with a remotely activated gate. When pigs were seen on camera inside the trap, the gate would be activated via a cellular network to close. They were then euthanized by using a small caliber rifle.

Whole blood samples (n = 276) were collected into 5 mL EDTA tubes while kidney (n = 315) and spleen (n = 51) samples were stored in Ziploc bags. All samples were transported on ice to the research lab within three hours of sample collection.

Serum samples were separated by centrifuging the whole blood at 1000× *g* for 10 min, and 200 µL plasma were transferred to microcentrifuge tube and stored at −20 °C. Remaining blood samples were mixed by vortex, transferred to microcentrifuge tubes and stored in −20 °C for nucleic acid extraction. A small portion of kidney and spleen tissues weighing approximately 200–300 mg was transferred to a microcentrifuge tube containing −800 µL of RNA/DNA stabilization buffer (Roche Diagnostics, Indianapolis, IN city, USA), and stored in −20 °C until nucleic acid extraction.

### 4.3. Collection of Environmental Samples

A total of 97 environmental samples (stream water and soil sediments) were collected during three sampling events, including 77 from the POL and 20 from the TNF. A drought during the summer of 2019 halted the collection of soil and water samples until stream flow increased at the sampling locations. Sampling at the properties occurred within 24 h of each other.

A wide-mouth collection bottle was rinsed in stream water before collection of a 500 mL grab sample. A soil auger was used to collect soil core samples to an approximate depth of 20 cm, which were placed in Whirl-Pak bags. Two water samples and three soil samples were collected at each sampling site and combined into one water and one soil sample per watershed. All samples were kept on ice while in the field and at the laboratory.

Upon return to the laboratory, 50 mL of water from each composite sample was pipetted into 50 mL sterile plastic test tubes. For each composite soil sample, 5 g of soil and 40 mL of double-distilled water were added to a 50 mL sterile plastic test tube.

Samples were vortexed at maximum speed for 2 min. Then, the environmental samples were centrifuged at 100× *g* for 5 min. The supernatant (30–35 mL) was recovered and centrifuged at 12,000× *g* for 20 min at room temperature. The supernatant was discarded, pellet was recovered, re-suspended in 1.5 mL of sterile double distilled water and centrifuged at 12,000× *g* for 20 min. Finally, the supernatant was discarded and pellet were stored at −20 °C until nucleic acid extraction.

### 4.4. Extraction of Nucleic Acids

The High Pure PCR Template Kit (Roche Diagnostics, Indianapolis, IN, USA) was used to extract nucleic acids in this study from whole blood, kidney and spleen, and environmental samples as described [39].

Whole blood (400 µL) was mixed with 400 µL of binding buffer followed by homogenization. For kidney and spleen, 200–300 mg of samples were mixed with 800 µL of RNA/DNA stabilization buffer and homogenized using 5 zirconia beads in a tissue homogenizer. For environmental samples, pellets were homogenized in 400 µL of binding buffer using 5 zirconia beads. A homogenate aliquot of 100 µL was mixed with 300 µL of binding buffer. After digestion with proteinase K (10% of total volume), extraction was completed following the established laboratory protocol as described [39]. Nucleic acid was eluted in the final volume of 200 µL using two step collection.

### 4.5. Molecular Detection of Leptospira spp. and DNA Sequencing

Two previously published PCRs [35,36] were used to identify *Leptospira* spp. in pigs and environmental samples. PCR was performed on a Roche Light Cycler 480 II Thermocycler. The primers and probes for *Leptospira* FRET-qPCR and the primers for *Leptospira* SYBR Green PCR were synthesized by Integrated DNA Technologies (Coralville, IA, USA) as described [35,36]. Ten µL of the extracted DNA was added to a 10 µL reaction mixture containing 5× PCR FRET buffer, 400 µM dNTP (Roche Diagnostics GmbH, Indianapolis, IN, USA), 0.34 units of Platinum Taq DNA Polymerase (Invitrogen, Waltham, MA, USA), 1 µM of each forward and reverse primer (Integrated DNA Technologies, Coralville, IA, USA) and a final volume of molecular grade nuclease-free water. PCR products for all positive and questionable samples were sent to ELIM Biopharmaceuticals (Hayward, CA, USA) for DNA sequencing using both ends of the primers, followed by BLASTn to identify the *Leptospira* species. The nucleotide sequences were submitted to NCBI to obtain GenBank Accession numbers.

### 4.6. Anti-Leptospira spp. IgG ELISA

The anti-*Leptospira* IgG antibody in pig plasma was measured by using the Porcine *Leptospira* IgG ELISA Kit (Novateinbio, Woburn, MA, USA) following the manufacturer’s instructions. A positive control, cut-off control, negative control, and substrate blank were included on each plate, and an optical density (OD) was measured at 450 nm with reference wavelength of 620 nm. Positive samples were interpreted as OD value higher than that of the cut-off OD value in each assay. The run validation and inter-assay precision was verified for its uniformity within range, using the similar OD values for controls, substrate blanks, and repeat testing of a select few samples.

### 4.7. Statistical Analysis

A chi-squared test was performed to compare the prevalence of anti-*Leptospira* IgG antibody-positive serum samples from pigs trapped in different months. Using bootstrapping, the 95% confidence intervals were calculated with the Statistics Base option in IBM SPSS Statistics for Windows, version 24.0 (IBM Corp., Armonk, NY, USA).

## Figures and Tables

**Table 1:** *Leptospira*-positive kidney tissue, environmental samples and the paired ELISA results.

Specimen Type	Sample ID	Sampling Dates	Species Identified by PCR	Anti-*Leptospira* IgG ELISA (OD Value)
Kidney	EAPL-1-2	July 19	*L. interrogans*	2.7
	EAPL-5-7	July 19	*L. interrogans*	1.36
	EAPL-25-5	August 19	*L. interrogans*	1.33
	EAPL-30-18	August 19	*L. noguchii*	1.57
	EAPL-30-20	August 19	*L. noguchii*	1.18
	EAPL-50-4	August 19	*L. noguchii*	2.15
	EAPL-20-5	September 19	*L. noguchii*	2.29
	EAPL-36-12	September 19	*L. noguchii*	3.22
	EAPL-25-17	November 19	*L. interrogans*	1.23
	EAPL-5-4-9	January 20	*L. noguchii*	1.21
Soil sediment	EP-2B-8	July 2019	*L. noguchii*	Not applicable
Stream water	Tusk-1W	July 2019	*L. interrogans*	Not applicable

**Table 2:** Temporal distribution of anti-*Leptospira* IgG antibody-positive serum samples.

Sampling Time	Total Assayed (N)	Positive (N)	Positivity (Confidence Interval)
July 2019	61	37	60.6% ^a^ (48.4%, 72.9%)
August 2019	35	20	57.1% ^a^ (40.8%, 73.5%)
September 2019	54	26	48.1% ^a^ (34.8%, 61.5%)
October 2019	46	16	34.8% ^ab^ (21.0%, 48.6%)
November 2019	16	5	31.2% ^ab^ (8.5%, 54.0%)
December 2019	14	5	35.7% ^ab^ (10.6%, 60.8%)
January 2020	25	9	36.0 ^ab^ (17.2%, 54.8%)
February 2020	7	2	28.6% ^ab^ (8.2%, 64.1%)
March 2020	18	3	16.7% ^b^ (5.8%, 39.2%)

Numbers followed by different letters (a, b) indicate significant differences between the positivity of *Leptospira* IgG antibody in different months of sampling times (Chi-squared test; *p* < 0.05).
